# A combinatorial therapy for treating obesity

**Published:** 2014-01

Obesity is quickly becoming a worldwide epidemic, and there are currently no effective therapies. Despite their promise, early treatment strategies failed to demonstrate clinical efficacy, which has prompted researchers to focus on the development of combinatorial therapies. Some of the earlier strategies relied on stimulation of β3-adrenergic receptors, and there is recent evidence that the effect of β3-adrenergic activation could be potentiated by combining it with PPARβ-receptor activation. To explore this possibility *in vivo*, Fernando Rodríguez de Fonseca and colleagues subjected rats to co-treatment with a β3-adrenergic agonist and a PPARβ-activating ligand. This had an effect on multiple processes involved in energy balance, including feeding, thermogenesis and fatty acid oxidation, promoting an overall increase in energy expenditure and decrease in energy intake. These results suggest that pharmacological combination of PPARβ and β3-adrenergic receptor agonists could represent a promising therapy for treating obesity. Page 129

